# Correction: Humans adapt their anticipatory eye movements to the volatility of visual motion properties

**DOI:** 10.1371/journal.pcbi.1008385

**Published:** 2020-10-21

**Authors:** The PLOS Computational Biology Staff

Figs 2 and 3 images are swapped in the manuscript, though the legends are correct. The correct image for Figs 2 and 3, along with the relevant legends, are provided here. The publisher apologizes for the error.

**Fig 2:**
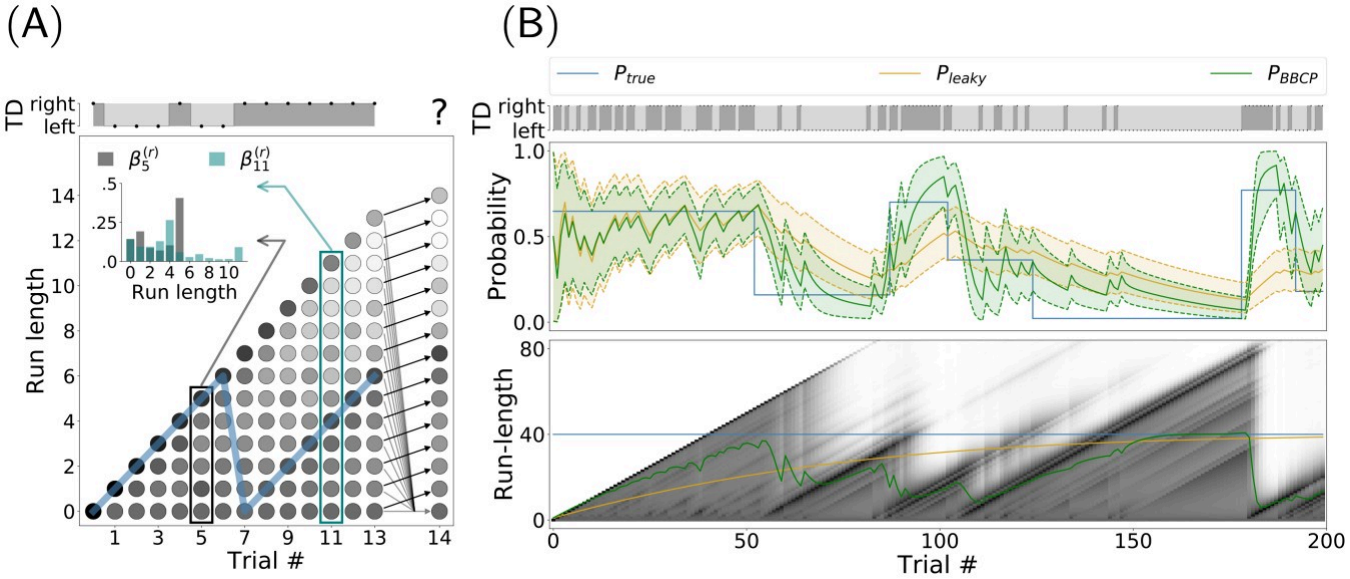
Binary Bayesian Change-Point (BBCP) detection model. (*A*) This plot shows a synthesized sequence of 13 events, either a leftward or rightward movement of the target (TD). Run-length estimates are expressed as hypotheses about the length of an epoch over which the probability bias was constant, that is, the number of trials since the last switch. Here, the true probability bias switched from a value of .5 to .9 at trial 7, as can be seen by the trajectory of the true run-length (blue line). The BBCP model tries to capture the occurrences of a switch by inferring the probability of different possible run-lengths. At any new datum (trial), this defines a Hidden Markov Model as a graph (trellis), where edges indicate that a message is being passed to update each node’s probability (as represented by arrows from trial 13 to 14). Black arrows denote a progression of the run-length at the next step (no switch), while gray lines stand for the possibility that a switch happened: In this case the run-length would fall back to zero. The probability for each node is represented by the grey scale (darker grey colors denote higher probability) and the distribution is shown in the inset for two representative trials: 5 and 11. Overall, this graph shows how the model integrates information to accurately identify a switch and produce a prediction for the next trial (e.g. for *t* = 14). (*B*) On a longer sequence of 200 trials, representative of a trial block of our experimental sequence (see Fig 1A), we show the actual events which are observed by the agent (TD), along with the (hidden) dynamics of the true probability bias *P*_true_ (blue line), the value inferred by a leaky integrator (*P*_leaky_, orange line) and the results of the BBCP model in estimating the probability bias *P*_BBCP_ (green line), along with .05 and .95 quantiles (shaded area). This shows that for the BBCP model, the accuracy of the predicted value of the probability bias is higher than for the leaky integrator. Below, we show the belief (as grayscales) for the different possible run-lengths. The green and orange line correspond to the mean run-length which is inferred, respectively, by the BBCP and leaky models: Note that in the BBCP, while it takes some trials to detect switches, they are in general correctly identified (transitions between diagonal lines) and that integration is thus faster than for the leaky integrator, as illustrated by the inferred value of the probability bias.

**Fig 3:**
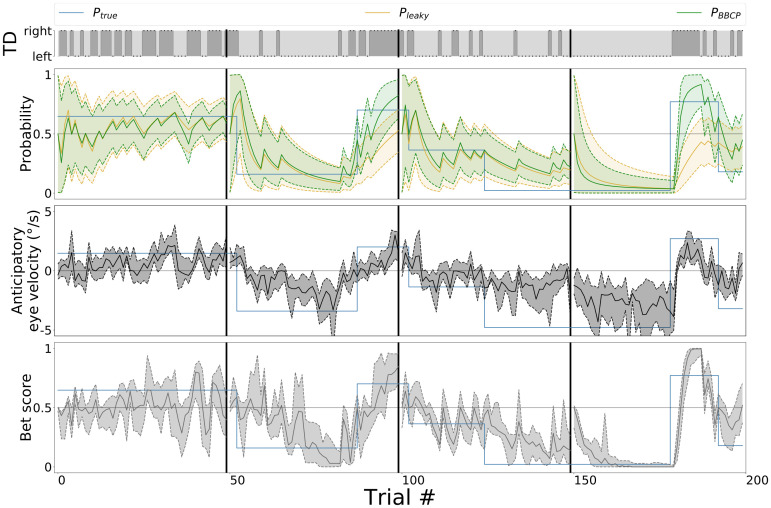
Behavioral results, qualitative overview. For one trial block of 200 trials, we compare the different model-estimated probabilities with respect to the behavioral results. The top row represents the sequence of target directions (TD) that were presented to observers and agents, as generated by the binary switching model (see Fig 1A). We show the evolution of the value of the (true) probability bias *P*_true_ (blue line) which is hidden to observers and that is used to generate the TD sequence above. We have overlaid the results of the probability bias predicted with a leaky integrator (*P*_leaky_, orange line) and with the BBCP model (*P*_BBCP_, see Fig 2B, green line). Bottom two rows display the raw behavioral results for the *n* = 12 observers, by showing their median (lines) and the .25 and .75 quantiles (shaded areas): First, we show the anticipatory pursuit eye velocity, as estimated right before the onset of the visually-driven pursuit. Below, we show the explicit ratings about the expected target direction (or *bet scores*). These plots show a good qualitative match between the experimental evidence and the BBCP model, in particular after the switches. Note that short pauses occurred every 50 trials (as denoted by vertical black lines, see main text), and we added the assumption in the model that there was a switch at each pause. This is reflected by the reset of the green curve close to the 0.5 level and the increase of the uncertainty after each pause.
