# Supplementary material for: Humans adapt their anticipatory eye movements to the volatility of visual motion properties
Source: PLoS Comput Biol. 2020 Apr 13;16(4):e1007438. doi: 10.1371/journal.pcbi.1007438 (PMC7179935; doi:10.1371/journal.pcbi.1007438)
Supplement: S1 Algorithm — (PDF) [file pcbi.1007438.s001.pdf]

# Supporting information to “Humans adapt their anticipatory eye movements to the volatility of visual motion properties”

Chloé Pasturel*, Anna Montagnini*, Laurent Udo Perrinet*†

Note: Equation numbers are those appearing in the main text. More information available at `https://github.com/laurentperrinet/PasturelMontagniniPerrinet2020`.

## 1 Appendix : leaky integrator

Given a series of observations $\{x_0^i\}_{0 \leq i \leq t}$ with $\forall i, x_0^i \in \{0, 1\}$, we defined

$$\begin{aligned}
\hat{x}_1^t &= (1 - 1/\tau)^{t+1} \cdot \hat{x}_1^{t=0} + 1/\tau \cdot \sum_{0 \leq i \leq t} (1 - 1/\tau)^i \cdot x_0^{t-i} \\
&= (1 - h)^{t+1} \cdot \hat{x}_1^{t=0} + h \cdot \sum_{0 \leq i \leq t} (1 - h)^i \cdot x_0^{t-i}
\end{aligned}$$

If we write it for trial $t - 1$, we have

$$\begin{aligned}
\hat{x}_1^{t-1} &= (1 - h)^t \cdot \hat{x}_1^{t=0} + h \cdot \sum_{0 \leq i \leq t-1} (1 - h)^i \cdot x_0^{t-1-i} \\
&= (1 - h)^t \cdot \hat{x}_1^{t=0} + h \cdot \sum_{1 \leq j \leq t} (1 - h)^{j-1} \cdot x_0^{t-j} \\
(1 - h) \cdot \hat{x}_1^{t-1} &= (1 - h)^{t+1} \cdot \hat{x}_1^{t=0} + h \cdot \sum_{1 \leq i \leq t} (1 - h)^i \cdot x_0^{t-i}
\end{aligned}$$

It follows that the integrative formula above becomes an iterative relation:

$$\begin{aligned}
\hat{x}_1^t &= (1 - h)^{t+1} \cdot \hat{x}_1^{t=0} + h \cdot \sum_{0 \leq i \leq t} (1 - 1/\tau)^i \cdot x_0^{t-i} \\
&= (1 - h)^{t+1} \cdot \hat{x}_1^{t=0} + h \cdot x_0^t + h \cdot \sum_{1 \leq i \leq t} (1 - h)^i \cdot x_0^{t-i} \\
&= h \cdot x_0^t + (1 - h) \cdot \hat{x}_1^{t-1}
\end{aligned}$$

*Institut de Neurosciences de la Timone (UMR 7289), Aix Marseille Univ, CNRS - Marseille, France
†Corresponding author:`Laurent.Perrinet@univ-amu.fr`

such that finally

$$\hat{x}_1^t = (1-h) \cdot \hat{x}_1^{t-1} + h \cdot x_0^t$$

As a result, the definitions in Equations (2) and (3)are equivalent.

# 2 The Bernoulli, binomial and Beta distributions

Let us define some basic concepts. A Bernoulli trial is the outcome of a binary random variable $x$ knowing a probability bias $\mu$ (with $0 \geq \mu \geq 1$) and can be formalized as:

$$Pr(x|\mu) = \mu^x \cdot (1-\mu)^{1-x}$$

The binomial distribution is defined as the probability that the sum $X$ of $\nu$ independent Bernoulli trials is $k$:

$$\Pr(k; \nu, \mu) = \Pr(X = k) = \binom{\nu}{k} \cdot \mu^k \cdot (1-\mu)^{\nu-k}$$

Knowing such a model for $X$, it can be of interest to find an estimate of the parameter of the Bernoulli trial, that is of the probability bias $\mu$. This distribution function is called the conjugate of the binomial distribution which is the Beta-distribution. For example, the beta distribution can be used in Bayesian analysis to describe initial knowledge concerning probability of success such as the probability that a product will successfully complete a stress test. The beta distribution is a suitable model for the random behavior of percentages and proportions.

It is usually defined using shape parameters $\alpha$ and $\beta$:

$$Pr(p|\alpha, \beta) = \frac{1}{B(\alpha, \beta)} \cdot p^{\alpha-1} \cdot (1-p)^{\beta-1}$$

Note that here, the variable is the probability bias $p$. The normalization constant $B(\alpha, \beta)$ is given by the beta function. By definition:

$$\alpha = \mu \cdot \nu$$
$$\beta = (1-\mu) \cdot \nu$$

Inversely, $\alpha + \beta = \nu$ and $\mu = \frac{\alpha}{\alpha+\beta} = 1 - \frac{\beta}{\alpha+\beta}$

# 3 Appendix 2: BBCP algorithm

To summarize, the algorithm that we presented is an implementation of the "Bayesian Online Changepoint Detection" by **?** extended for the class of binary inputs. Using the definition of the run-length (see Section "Binary Bayesian Change-Point (BBCP) detection model" in the main text), the flow-chart of the algorithm is:

1. Initialize
    - $P(r_0 > 0) = 0$ or $P(r_0 = 0) = 1$ and

- $\mu_0^{(0)} = \mu_{prior}$ and $\nu_0^{(0)} = \nu_{prior}$

2. Observe New Datum $x_0^t \in \{0, 1\}$,

   (a) Evaluate Predictive Probability $\pi_t^{(r)} = P(x_0^t | \mu_t^{(r)}, \nu_t^{(r)})$.

   (b) Calculate Growth Probabilities $P(r_t = r_{t-1} + 1, x_{0:t}) = P(r_{t-1}, x_{0:t-1})\pi_t^{(r)}(1-h)$,

   (c) Calculate Changepoint Probabilities $P(r_t = 0, x_{0:t}) = \sum_{r_{t-1}} P(r_{t-1}, x_{0:t-1})\pi_t^{(r)} \cdot h$,

   (d) Calculate Evidence $P(x_{0:t}) = \sum_{r_{t-1}} P(r_t, x_{0:t})$,

   (e) Determine run-length Distribution $P(r_t | x_{0:t}) = P(r_t, x_{0:t})/P(x_{0:t})$.

3. Update sufficient statistics

   - at a switch $\mu_{t+1}^{(0)} = \mu_{prior}$, $\nu_{t+1}^{(0)} = \nu_{prior}$,
   - else, $\nu_{t+1}^{(r+1)} = \nu_t^{(r)} + 1$ and $\nu_{t+1}^{(r+1)} \cdot \mu_{t+1}^{(r+1)} = \nu_t^{(r)} \cdot \mu_t^{(r)} + x_0^t$.

4. Return to step 2.

In the following, we detail some intermediate steps and highlight some key differences with their implementation. We also provide a python implementation of the algorithm, which is available at `https://github.com/laurentperrinet/bayesianchangepoint`.

## 3.1 Initialization

Note that the prior distribution is itself a Beta distribution: $\mathcal{P} \propto B(p; \mu_{prior}, \nu_{prior})$. It will by symmetry be unbiased: $\mu_{prior} = .5$. Concerning the shape, it can be for instance the uniform distribution $\mathcal{U}$ on $[0, 1]$, that is $\nu_{prior} = 2$ or Jeffrey's prior $\mathcal{J}$, that is $\nu_{prior} = 1$. We chose the latter for the generation of trials as the uniform distribution would yield more samples around $p = .5$. Qualitatively, this would result in more difficult task in discriminating a probability bias from another. Jeffrey's prior was more adapted to that task.

## 3.2 Prediction: run-length distribution

The steps to achieve the update rule are:

$$
\begin{aligned}
Pr(x_0^t | x_0^{0:t-1}) &= \sum_{r^t} Pr(x_0^t | r^t, x_0^{0:t-1}) \cdot \beta_t^{(r)} \\
Pr(x_0^t | x_0^{0:t-1}) &= \sum_{r^t} Pr(x_0^t | r^t, x_0^{0:t-1}) \cdot Pr(r^t | x_0^{0:t-1}) \\
\text{with} \quad Pr(r^t | x_0^{0:t-1}) &\propto \sum_{r^{t-1}} Pr(r^t | r^{t-1}) \cdot Pr(x_0^t | r^{t-1}, x_0^{0:t-1}) \cdot Pr(r^{t-1} | x_0^{0:t-2})
\end{aligned}
$$

Finally we obtain Equation (5):

$$
\beta_t^{(r)} \propto \sum_{r^{t-1}} Pr(r^t | r^{t-1}) \cdot Pr(x_0^t | r^{t-1}, x_0^{0:t-1}) \cdot \beta_{t-1}^{(r)}
$$

### 3.3 Prediction: sufficient statistics

The recursive formulation in Equations (9) and (10)comes from the expression

$$\nu_t^{(r)} \cdot \mu_t^{(r)} = \sum_{i=t-r-1}^{t-1} x_0^i$$

and therefore

$$\begin{aligned}
\nu_{t+1}^{(r+1)} \cdot \mu_{t+1}^{(r+1)} &= \sum_{i=t+1-r-1-1}^{t+1-1} x_0^i \\
&= \sum_{i=t-r-1}^{t} x_0^i \\
&= \nu_t^{(r)} \cdot \mu_t^{(r)} + x_0^t
\end{aligned}$$

### 3.4 Quantitative evaluation

To quantitatively evaluate our results with respect to another probability bias, we computed in Equation (13) the cost as the Kullback-Leibler divergence $\mathrm{KL}(\hat{p}|p)$ between samples $\hat{p}$ and model $p$ under the hypothesis of a Bernoulli trial:

$$\mathrm{KL}(\hat{p}|p) = \hat{p} \cdot \log\left(\frac{\hat{p}}{p}\right) + (1-\hat{p}) \cdot \log\left(\frac{1-\hat{p}}{1-p}\right). \tag{1}$$

# 4 Appendix: likelihood function

We want to compute $\mathcal{L}(r|o) = Pr(o|p, r)$ where $o \in \{0, 1\}$ such that we can evaluate Predictive Probability $\pi_{0:t} = P(x_0^t|\mu_t^{(r)}, \nu_t^{(r)})$ in the algorithm above with $\mu_t^{(r)}$ and $\nu_t^{(r)}$ the sufficient statistics at trial $t$ for node $(r)$. The likelihood of observing $o = 1$ is that of a binomial (conjugate of a Beta distribution) of

- mean rate of choosing hypothesis $o = 1$ equal to $\frac{p \cdot r + o}{r+1}$,
- number of choices where $o = 1$ equals to $p \cdot r + 1$.

More generally, by observing $o$, the new rate is $p^{'} = \frac{p \cdot r + o}{r+1}$.

### 4.1 Mathematical derivation

The likelihood will give the probability of this novel rate given the known parameters and their update (in particular $r^{'} = r + 1$):

$$
\begin{aligned}
L(r|o) &= (\frac{p\cdot r + o}{r+1})^{p\cdot r+o} \cdot (1 - \frac{p\cdot r+o}{r+1})^{r+o-(p\cdot r+o)} \\
&= \frac{1}{(r+1)^{r+1}} \cdot (p\cdot r+o)^{p\cdot r+o} \cdot ((1-p)\cdot r+1-o)^{(1-p)\cdot r+1-o}
\end{aligned}
$$

since both likelihood sum to 1, the likelihood of drawing $o$ in the set $\{0,1\}$ is equal to

$$
\begin{aligned}
\mathcal{L}(r|o) &= \frac{L(r|o)}{L(r|o=1)+L(r|o=0)} \\
&= \frac{(p\cdot r+o)^{p\cdot r+o} \cdot ((1-p)\cdot r+1-o)^{(1-p)\cdot r+1-o}}{(p\cdot r+1)^{p\cdot r+1}\cdot((1-p)\cdot r)^{(1-p)\cdot r} + (p\cdot r)^{p\cdot r}\cdot((1-p)\cdot r+1)^{(1-p)\cdot r+1}} \\
&= \frac{(1-o)\cdot(p\cdot r)^{p\cdot r}\cdot((1-p)\cdot r+1)^{(1-p)\cdot r+1} + o\cdot(p\cdot r+1)^{p\cdot r+1}\cdot((1-p)\cdot r)^{(1-p)\cdot r}}{(p\cdot r+1)^{p\cdot r+1}\cdot((1-p)\cdot r)^{(1-p)\cdot r} + (p\cdot r)^{p\cdot r}\cdot((1-p)\cdot r+1)^{(1-p)\cdot r+1}}
\end{aligned}
$$

This can also be written by isolating the part which depends on $o$ and for a given run-length and knowing sufficient statistics describing the sufficient statistics at each node $r$:

$$
\mathcal{L}(r|o) = \frac{1}{Z}\cdot(p\cdot r+o)^{p\cdot r+o}\cdot((1-p)\cdot r+1-o)^{(1-p)\cdot r+1-o} \tag{2}
$$

with $Z$ such that $\mathcal{L}(r|o=1)+\mathcal{L}(r|o=0)=1$, that is Equation (11).

## 4.2 Python code

```
def likelihood(o, p, r):
    """
    Knowing $p$ and $r$, the sufficient statistics of the beta distribution $B(\alpha,
        \beta)$ :
    $$
        alpha = p*r
        beta  = (1-p)*r
    $$
    the likelihood of observing o=1 is that of a binomial of

        - mean rate of choosing hypothesis "o=1" = (p*r + o)/(r+1)
        - number of choices where "o=1" equals to p*r+1

    since both likelihood sum to 1, the likelihood of drawing o in the set {0, 1}
    is equal to

    """
    def L(o, p, r):
        P =  (1-o) * ( 1. - 1 / (p * r + 1) )**(p*r) * ((1-p) * r + 1)
        P +=  o * ( 1. - 1 / ((1-p) * r + 1) )**((1-p)*r) * (p * r + 1)
```

```
    return P

L_yes = L(o, p, r)
L_no = L(1-o, p, r)
return L_yes / (L_yes + L_no)
```

See the code online at `https://github.com/laurentperrinet/bayesianchangepoint`.

### 4.3 Properties

This function has some properties, notably symmetries:

- for certain outcomes, $\forall r > 0$, $\mathcal{L}(o|p=0,r) = 1-o$ and $\mathcal{L}(o|p=1,r) = o$,
- if $r = 0$, the likelihood is uniform $\mathcal{L}(o) = 1/2$,
- $Pr(o|p,r) = Pr(1-o|1-p,r)$.

Note also that as $r$ grows, the likelihood gets sharper.
