# Supplementary material for: Humans adapt their anticipatory eye movements to the volatility of visual motion properties
Source: PLoS Comput Biol. 2020 Apr 13;16(4):e1007438. doi: 10.1371/journal.pcbi.1007438 (PMC7179935; doi:10.1371/journal.pcbi.1007438)
Supplement: Attachment — Submitted filename: Reviews_BCCP-3.pdf [file pcbi.1007438.s002.pdf]

Response to reviewers

We are grateful to the reviewers for their encouraging remarks and useful comments which were essential in improving our manuscript. In addition to the revised manuscript, we detail here our response to the different points being made while showing some screenshots of the revised manuscript when useful. A "revised tracked changes" is also available as a supplementary file to see for all changes being made since the initial revision.

Reviewer #1:

The authors use anticipatory pursuit eye movements and explicit judgements to study how humans adapt to the volatility of their environment (i.e. how the probability of specific events changes; foraging for food could give an ecologically relevant example). Whereas anticipatory pursuit and other behaviours have been studied rather extensively to investigate the integration of probability in decision-making there has been little attention being paid to higher-level statistics. The authors put forth a Bayesian model for explaining adaptation to the latter, extending considerably the complexity of current models. The experimental paradigm is a simple task in which the observer sees leftward and rightward motion of a dot with varying probabilities. Those probabilities are fixed within a block of trials and this probabilities change at random run lengths (unbeknown to the participant).

The article centres upon contrasting a new Bayesian model predicting probability transitions to a simple leaky integrator model. They then compare the appropriateness of the model in explaining behaviour (anticipatory pursuit, i.e. the supposedly implicit eye movement that anticipates target motion, and the explicit "bets" on whether the target moves leftward or rightward).

I find the paper an interesting addition to the rather vast literature on sequence learning with implicit and explicit measures. The authors show indeed that the Bayesian model outperforms the leaky integrator model, providing a useful and novel description of sequence learning.

We thank the reviewer for these positive comments

Major points

I feel the writing could be much more concise and terms could be used more consistently. For instance, lines 773-809 of the discussion seem entirely gratuitous to me. Overall, the readership may be put off by repetition and at times an overly abstract argumentation.

We thank the reviewer to point out this weakness of our manuscript and have made a consistent effort to shorten, simplify and reduce redundancy in the prose at several places. The most important changes are listed here and all changes are highlighted in the tracked changes PDF. Main points are:

1) *We removed many redundant parts, in particular those mentioned by the reviewers' minor points;*
2) *Regarding the theoretical section about the implementation of hierarchical Bayesian inference in the brain (previous lines 773-809), we respectfully disagree with the reviewer about the lack of importance of this part, as the strengths and the limits of this theoretical approach are indeed strongly debated in the brain science community, and it is important to be clear on these issues. Nevertheless, we realize that in the former version, the message was rather "sluggish" and the text (way) too long. We have now made this text more concise and hopefully more direct to the point:*

When we perceive the physical world, make a decision or take an action to interact with it, our brain must deal with an ubiquitous property of it, uncertainty. Uncertainty can arise at different levels and be structured around different characteristic time scales. The theoretical framework of Bayesian probabilistic inference, which provides a formal account for the role of uncertainty at multiple levels, has become very popular as a benchmark of optimal behavior in perceptual, sensorimotor and cognitive tasks [74] and, more generally, as a unified framework for studying the brain [75]. Importantly,

plausible hypotheses about the implementation of Bayesian computations —or approximations of them— in the activity of neuronal populations have been proposed [76–78]. However, one should be careful when evaluating the quality of fit of Bayesian inference models for behavioral data, and avoid any over-interpretation of the results. This kind of model fitting aims at evaluating the adequacy of a specific generative inferential model, not of the probabilistic calculus in its detailed implementation. Still, there is actually a common confusion around the idea of a "Bayesian brain", and we believe that the challenge here is not to validate the hypothesis that the brain implements or not the Bayes' theorem, or a more complex hierarchical combinations of inferential computations, but rather to test hypotheses about the different generative models that agents may use.

I also feel that the mathematical explanation of the model could be made clearer, perhaps by using different symbols rather than superscript to differentiate between different estimates.

The mathematical notation inherits from previous work (notably Adams and MacKay (2007) and Wilson et al (2013)) and we have kept these notations to highlight our contributions. We have tried to enhance our description to improve the readability.

While the discussion lacks concision, on the other hand the authors take little stock of their findings. For instance, the lack of correlation between explicit and implicit measures when it comes to estimates of parameter h (a very similar point was made here: https://www.frontiersin.org/articles/10.3389/fnhum.2016.00227/full), which they call hazard rate.

We thank the reviewer for highlighting this point. We definitely agree that this is one of the most (somewhat unexpected) interesting results that deserve further investigation. In addition to simplifying the corresponding Figure in response to a comment of the other Reviewer, we have added a new sentence in the discussion to

Results Section 4:

Second, there is an apparent lack of correlation between the explicit and the implicit estimates of the hazard rate, yet we would need more empirical evidence to prove that this originates from the experimental setup or rather by separate processing of volatility. Such an analysis would suggest that even though the predictive processes at work in both sessions may reflect a common origin for the evaluation of volatility, this estimation is then more strongly modulated by individual preferences when a more explicit cognitive process is at stake.

and further:

Future work might provide important insight about the analysis of these inter-individual differences in terms of each participant's preference for exploration versus exploitation across different cognitive tasks.

We further thank the reviewer for mentioning the interesting work by Souto, Gegnfurtner and Schuetz, which is now also included in the reference list. However, we would like to precise that there is an important difference in the kind of behavioural measures (the implicit and explicit ones) compared in that study and in ours. In that work, authors found a lack of correlation between the saccadic adaptation rate and the inferred estimation of uncertainty in terms of JND extracted from the psychometric curves. In our view the latter cannot really be considered as an individual "explicit" estimate of uncertainty, at least not at the same level as our "explicit rating" measures of the certainty attributed to the expected motion direction of the forthcoming moving target. The focus, here, is on whether implicit (anticipatory eye movements) and explicit (ratings) individual estimates of uncertainty undergo similar and correlated dynamic changes across the trial sequence; in the cited study, the

focus was rather on the correlation between the condition-averaged magnitude of perceptual uncertainty and the dynamics of oculomotor adaptation.

We have thus reorganized and incremented a paragraph in the Discussion (lines 773 -788) to more thoroughly address the relation between implicit and explicit estimates of the probability bias, and include some references to previous work.

The distinction between implicit and explicit processes in the adaptation to a volatile environment has also been addressed by previous work, especially in the field of statistical learning for language processing (see for example [69,70]). More related to the present study, Wu et al [71] compared a classical economic decision task with a motor decision task: they found that participants were more risk seeking in the motor task compared to the first one. In addition, Souto et al [72] have recently reported a lack of correlation between the rate of oculomotor adaptation to unexpected jumps of the visual target and the perceptual uncertainty estimated through an explicit jump direction-discrimination experiment. Finally, the degree of explicitness of the information provided to the participants may also play a role in the context of probabilistic learning. In a task similar to ours, where the behavioral choice was not specifically associated to a reward schedule, Santos and Kowler [51] found large similarities but also some differences in the anticipatory eye movements depending on how the information about the probability bias was conveyed, namely through the simple presentation of a biased sequence versus an explicit probability-cueing procedure.

Also, I was expecting some discussion of something that is rather conspicuous in Figure 4A. The eye movements seem to match the true probability better than both the leaky and Bayesian model. It would seem that the Bayesian model doesn't fare well to explain the eye movements. I am then left wondering what further information the user can use to beat the optimal observer?

As a matter of fact, eye movements match better the Bayesian model than the leaky integrator or the true probability. This is put forward by the statistics on the r2 coefficients as well as on the mutual information, but was not evidenced by the plot. We have enhanced the presentation of the statistics (on n=12 subjects) and improved the presentation of the plot.

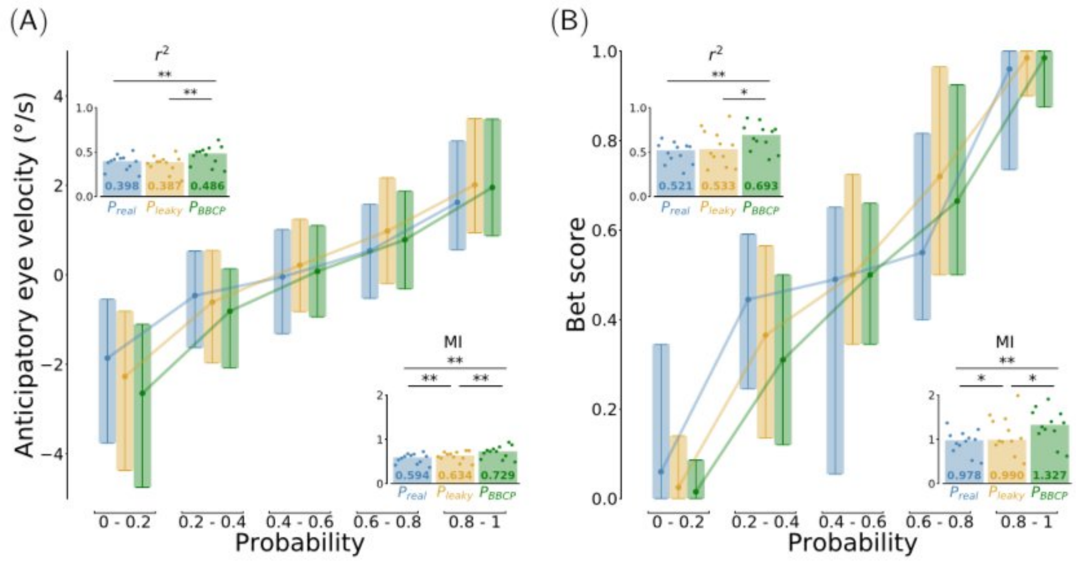

Fig 4. Behavioral results, quantitative analysis across participants ($n = 12$).

It seems odd not to cite the work of Collins and Barnes on anticipatory pursuit with different probability blocks: https://www.jneurosci.org/content/29/42/13302.s hort

We agree with the reviewer that Collins and Barnes, and more in general the group of Graham Barnes, have provided an invaluable contribution to the literature on the predictive control of eye movements, although they have not -to our knowledge- specifically addressed either the role of probability (they rather focused on the central tendency of the recent trial history), or the contextual volatility. We also agree that in the previous version of the manuscript we had not appropriately cited their work. In addition to the suggested article (« Predicting the Unpredictable: Weighted Averaging of Past Stimulus Timing Facilitates Ocular Pursuit of Randomly Timed Stimuli »), we have added another appropriate and due citation of a review by the same author at lines 151 (see also below for a point on the similar line).

> appearance [32–34] thereby reducing visuomotor latency [35]. Moreover, some experiments have demonstrated the existence of prediction-based smooth pursuit maintenance during the transient disappearance of a moving target [36–38] and even predictive acceleration or deceleration during visual tracking [37, 39]. Overall, although the initiation of smooth pursuit eye movements is almost always driven by a visual motion signal, it is now clear that smooth pursuit behavior can be modulated at different stages by extra-retinal, predictive information even in the absence of a direct visual stimulation [40]. Several functional and computational models have been

Regarding the mathematical interpretation I am not sure that hazard rate is a good term for the term h, which in a leaky-integrator model is more known as the leaking rate or forgetting rate. The hazard rate refers to something different. I am not sure whether I am missing something.

We have kept the term "hazard rate" throughout the text as it calls to the cause of the volatility in the Bayesian model, as the probability at every trial to observe a switch. Indeed, this translates phenomenologically to the forgetting rate of the leaky-integrator and one of our goals is to more rigorously make the link between the two variables. This has in particular been highlighted in Section 2.

Minor and more specific comments

A matter of taste perhaps: Acronyms often save time for the writer but not the reader, also I find aSPEM are rather ugly-sounding acronym. "anticipatory pursuit", isn't much longer.

Indeed, this sounds better and as short and we have replaced occurrences of the acronym when possible.

lines 61-63 the link to the measles outbreak could be made explicit.

Done, thanks.

Consider removing needless words: "As such,", "in all generality," ...

We have improved the overall readability and had the manuscript intensively proofred. Please see the tracked changes' PDF that highlights all the changes we have done on the manuscript.

Paragraph 76-91 is rather obscure to me, it requires some more explanation. The link to adaptation and priming is probably misleading as the measure corresponds rather to a prediction of what happens next, those two effects are typically tested differently (a reduction of sensitivity and an increase in sensitivity)

We have written anew this paragraph to highlight the current understanding of adaptation effects in psychophysics and in particular to show how an integrated approach such as the Bayesian model that we propose helps to understand these in an unified manner. See our changes to the text at lines 71-87 :

In controlled psychophysical experimental settings which challenge visual perception or sensorimotor associations, such adaptive processes have been mostly put in evidence by precisely analyzing the participants' behavior in a sequence of experimental trials. These typically highlight sequential effects at the time scale of several seconds to minutes or even hours in the case of the adaptation to a persistent sensorimotor relation. Indeed, stimulus history of sensory events influences how the current stimulus is perceived [3–7] and acted upon [8–11]. Two qualitatively opposite effects of the stimulus history have been described: negative (adaptation), and positive (priming-like) effects. Adaptation reduces the sensitivity to recurrently presented stimuli, thus yielding a re-calibrated perceptual experience [12–14]. On the other hand, priming is a facilitatory effect that enhances the identification of repeated stimuli [15, 16]: in sensorimotor control, the same stimulus presented several times could indeed lead to faster and more precise responses. Interestingly, priming effects are sometimes paralleled by anticipatory motor responses which are positively correlated with the repetition of stimulus properties. A well-known example of this behavior are anticipatory smooth eye movements (aSPEM or shortly, anticipatory pursuit), as we will illustrate in the next section.

line 79: "yielding a"

Done, thanks.

line 82: not clear how adaptation "favors spatial stability of the stimulus"

We have removed the spatial aspect of adaptation as it is unrelated to our study.

line 86: quite obscure

This was cut with the change of the paragraph in which the line was enclosed.

The statement line 145 needs to be substantiated. I am not aware of a study testing this that anticipatory pursuit is unconscious, although work on saccades (capture) would suggest so based on the time-scales involved. This points appears rather central in the motivation of the study (contrast implicit and explicit mechanisms), therefore it needs more consideration.

The reviewer is right: the lack of awareness about one's own anticipatory smooth eye movements has not really been demonstrated in a rigorous way and it is largely based on informal exchanges with participants (and introspection). We have now eliminated the reference to any comparison of conscious vs unconscious measures, whereas we maintain and discuss the distinction between explicit vs implicit measures, although we do not aim at providing any strong categorization with this dichotomy here. Anticipatory eye movements are referred to as "implicit" here, simply in contrast to the clearly "explicit" nature of the rating. We have now strongly nuanced the above-mentioned statement and associated it to another one focusing on a more pragmatic point:

timing or direction [10, 44, 46]. Second, it is a robust phenomenon, which has been observed on a large population of human participants and even in non-human primates (for a recent review see [47]). Note also, that human participants seem to be largely unaware of this behavior (as inferred from informal questioning). Finally, this kind of behavior has proven to be exquisitely sensitive to the probabilistic properties of the sensorimotor context.

In addition, we have shortened the whole section 1.2 as it was redundant at several places.

line 160: coherent "with"

Done, thanks.

line 192: Is it meant to be "volatility" and not "variability"?

Indeed, this was not clear and we changed the formulation :

> a given diffusion coefficient). Ultimately, this hierarchical model allows to generate a sequence of binary choices where volatility is controlled by a specific random variable which fluctuates in time according to some probabilistic law. Such a forward

line 195-197 the term trial block may lead to some confusion, as it is meant to be the sequence of trials with the same probability but also commonly the number of trials in a full sequence.

We have tried to disambiguate this terminology by using consistently "epoch" for a block of trials between two successive switches and "trial block" for a block between successive pauses.

line 318: the reference should be included when you first mention this heuristic, otherwise it feels like you refer to a new one.

Done, thanks.

I can't make much sense of 324-326. The point that Equation 3 assumes a constant weight is also obscure to me. Doesn't a leaky-integrator amount to weighting past trials depending on trial number? This needs to be clarified.

Indeed, this was not clear and we changed the formulation:

> In particular, the free parameter of the model (h), may be fitted to the behavioral dataset. Testing different hypothesis for the value of h thus allows to infer the agents' most likely belief in the (fixed) weight decay. Now, since we have defined a first

Figure 2: I take the blue line in A to represent the black lines being mentioned.

Fixed, thanks.

Fig 4. An indication of n on the figure would be welcome (I assume it is n observers). "For all participants and for all trials" is an ambiguous description. Was it one estimate per participant, averaging all trials? or were all trials pooled together disregarding participants?

We have modified the captions of each figure to indicate the number of participants and to give more precision on the way we performed the statistics.

Oddly I couldn't find a definition of an interval over which anticipation was measured for the eye movements. What was the averaging interval? In the same vein, the goal of the fitting procedure is unclear to me. Why not average eye movemnets during an time-window corresponding to a period before visual information kicks in?

We have now clarified this important point (indeed, we do not define an arbitrary fixed temporal window of anticipation), and we have more precisely explained in the text the method used to estimate the anticipatory eye velocity, in comparison to a more classical method:

> steady-state. This analysis was applied to each trial individually and it allowed in particular to estimate the velocity of anticipatory pursuit as the best-fit value of the modeled eye velocity at the moment where the visually-guided pursuit is initiated. Note

This procedure is illustrated below (but we think that in the interest of being concise this figure should not be added to the manuscript) and the Reviewer can also appreciate for this example trial the general model fitting performance and the kind of parameters we estimated. Note that this method for estimating anticipatory velocity led to qualitatively identical results to the estimation of the mean eye velocity within an arbitrary temporal

window of anticipation, a more classical method that we implemented for instance in a previous study (Damasse et al. 2018)

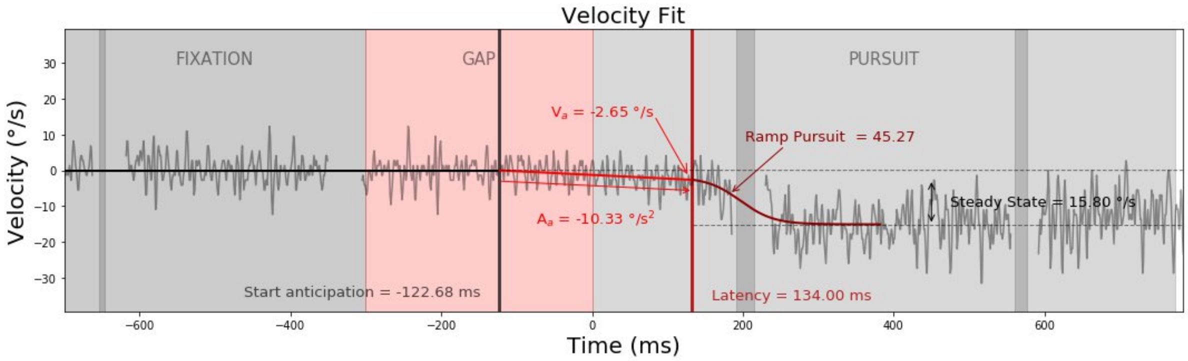

Figure 5 is quite confusing. It is trying to show too much. It would be more important to highlight the main conclusions, such as the comparison to ground truth, and the lack of correlation between h in the bet and spem estimate.

Indeed, this figure was overlying too much information and we have simplified it to show all the data in a simpler way:

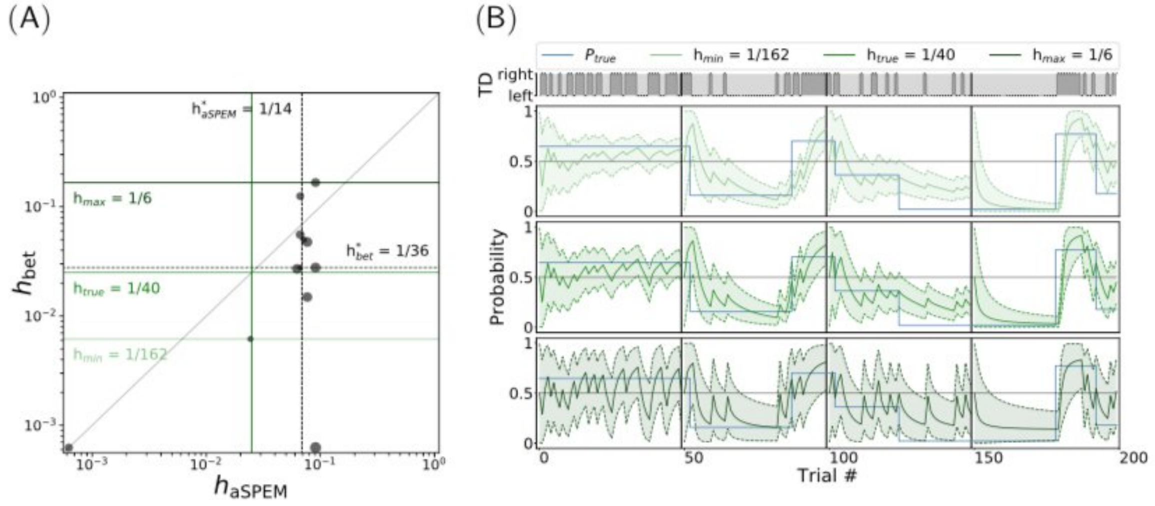

Fig 5. Analysis of inter-individual differences. *(A)* We analyzed the behavior of

Line 606 to 616 this seems more like a discussion point. Also I am not clear how one of the statements is tested, regarding the fact variability in the estimate scales with inferred probability.

Indeed, we moved this part to the discussion, which allowed to remove some redundancies.

Reviewer #2:

The authors presented a white ring that could move leftward or rightward. In one of the

experimental sessions, the participants used smooth pursuit eye movements to track the motion of the ring. In a second experimental session, the participants were asked to “bet” or guess the direction of motion by placing a cursor on a continuous rating scale. The anticipatory smooth eye movements of the participants were modeled using a forgetful agent and a Bayesian model agent. The quantitative analysis of the authors showed close agreement between their Bayesian model agent and the participants’ anticipatory smooth eye movements and ratings.

Some of the strengths of this manuscript is that they made reasonable assumptions for their models. I was able to follow the logic behind the formulas and assumptions that they made. Directly comparing and modeling unconscious and conscious motor anticipatory behavior is a relatively novel and an important contribution. However, there are a few parts of the manuscript that need to be clarified.

We thanks reviewer #2 for his encouraging and valuable comments. In our revision, we have tried to put forward these strong points and render them more visible to the readers. Let us only precise that we prefer to refer to our two experimental measures (anticipatory pursuit and confidence ratings) as implicit and explicit respectively, rather that unconscious and conscious, as we do not have a rigorous assessment of the unconscious nature of anticipatory pursuit. This point is also the object of an observation of Reviewer 1.

Figure 3 needs to be reworked or the data needs to be presented differently. The way it is presented in the manuscript is too complicated. It took me too long to figure out what all of the lines mean, and I’m still not sure that I understand what the figure is showing. I read what the manuscript says about this figure and it may make sense, but it is difficult to see it in graph. I’m not saying that they authors are wrong or right; I just can’t understand what’s going on in the figure. Perhaps the authors can move this figure to the supplemental material and just show a simplified version of this figure in the manuscript. What does the negative and positive velocities indicate? Direction of motion?

Indeed, Figure 3 synthesizes in one figure all traces: the probability-bias implemented following the generative model, individual ratings, anticipatory eye velocity and Bayesian model predictions. In the revised version, while keeping the overall architecture for the figure, we have tried to make it more readable and tried to improve its caption. Instead of showing the stacking of all modalities for two subjects (with one panel per subject), we now show one panel per modality (probability-bias / anticipatory pursuit / rating) for the whole group of n=12 participants. This better illustrates the methodology in the paper which confronts experimental data with theoretical predictions:

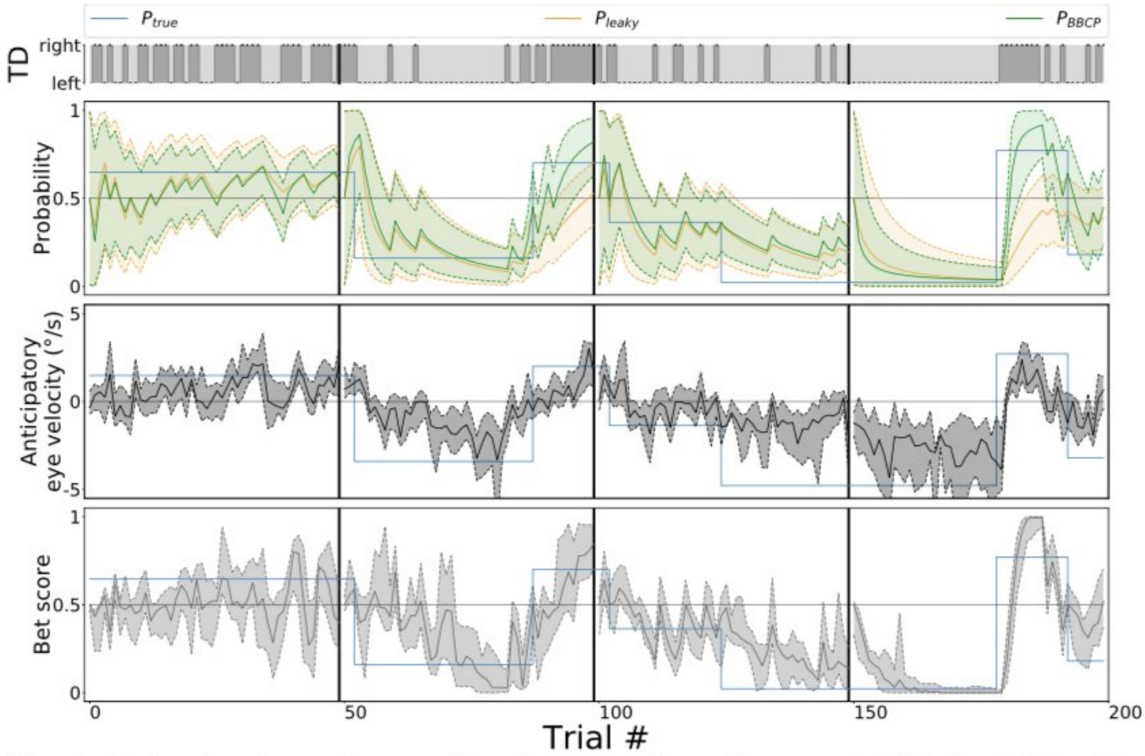

Fig 3. Behavioral results, qualitative overview. For one trial block of 200 trials,

I also had trouble following what's going on in Figure 5. Can this figure have another panel showing the anticipatory smooth eye movements (gains?) the authors get with their paradigm for each individual subject? Or maybe just have another figure showing if there are any sequential effects? What does the anticipatory smooth eye movements look like when there is no switch for a relatively large number of trials? Is there a relationship between participants who tended to be more sure/confident about their assumption of the upcoming direction of motion? Do these results agree or disagree with previous studies?

Similarly, Figure 5 was cluttered with too much information (see also the comment from reviewer #1). We have simplified it by showing the analysis (estimated hazard rates) for each individual participant. Last, we have added a panel showing the response of the model for three characteristic levels of the hazard rate and corresponding to the range of volatilities observed across participants.

Another part of the manuscript that can be clearer is the paradigm of the experiment. Starting at line 863, the manuscript says, "the moving target, which was presented slightly offset from the fixation location and immediately started moving horizontally at a constant speed of 15°/s, either to the right or to the left for 1000 ms." Can you clarify this? Is this a "step-ramp"? Or, is it that the target appears off center and then begins to move? I was wondering if some of the participants of the study could have noticed the slight offset and used it as a cue for the direction of motion. For example, white ring is slightly to the left of the fixation cross therefore it will move toward the right. I know of at least one study that has used location of a cross as a cue to direction of motion for anticipatory smooth eye movements. If this is the case, the authors may need to disentangle what is anticipatory smooth eye movements in response to past history or in response to a cue. Could this explain some of the individual differences that the authors found? I was also looking at how much bigger the error bar plots for the "Bet score" are in Figure 4 than the "velocity of eyes". Do the authors have any idea why this could be? I also didn't understand how "strength of aSPEM" was calculated. Why is the median being used (instead of the mean)?

We are grateful to the reviewer to have raised our attention to several points lacking clarity. We have now more precisely described the experimental trial design, and explicitly mentioned the step-ramp paradigm at lines 891-

894. As the initial target position is randomized across trials and not disclosed until the end of the gap, we can exclude that it is used as a cue for target motion.

fixation point and the onset of the moving target. The target was then presented slightly offset from the fixation location (*step-ramp* paradigm [86]), either to the right or to the left, and immediately started moving horizontally toward the center at a constant speed of $15°/s$, for 1000 ms. The probability p of rightward motion trials was a

With regard to the large error bars observed for the Bet experiments, it is definitely true that the inter-individual variability was larger for the explicit ratings than for the anticipatory velocity : interestingly this is true both for the raw measures (as indicated by the large error bars in Figure 4B) and for the inferred value of the hazard rate (as can be appreciated in the broad scatter of the vertical data-points in Figure 5). We can speculate that such large variability is possibly the result of a mixture of different cognitive processes that could be at play for the Bet experiment. For instance, some participants could be very conservative, and retain from large confidence ratings, while others could adapt their bet to the systematic count of Right- and Leftward moving targets during the few past trials.

Furthermore, we acknowledge that the use of the term "strength of aSPEM" was confusing, and we have now replaced almost all occurrences of the term "strength" by the more appropriate "anticipatory pursuit velocity" (note that we have now modified also our notation for aSPEM, following Reviewer 1's suggestion, and refer now to "anticipatory pursuit"). Having said that, we are now hopefully more clearly defining the anticipatory pursuit velocity as the best-fit estimated eye velocity right before visually guided eye movements onset (see response -and illustration- to one of Reviewer 1' s minor comments).

Finally we use of the median instead of the mean as in general the behavioral data is not Gaussian and therefore the median provides with a more robust statistical descriptor.

The manuscript would also benefit from adding a paragraph briefly describing the progression/evolution of models of anticipatory smooth eye movements and how their model will fit (agree or disagree) with existing models.

We appreciate this suggestion by the reviewer and we have now added a small paragraph (lines 136-146) briefly addressing the few attempts, in the literature, to provide a theoretical framework for anticipatory smooth pursuit. Note, however, that the novelty of our Bayesian model lies in the fact that it takes into account inferential knowledge about the volatility of the environment, rather than in the hypotheses about the link between such inferential knowledge and anticipatory smooth pursuit.

visual stimulation [40]. Several functional and computational models have been proposed in the literature for the different forms of prediction-based smooth eye movements, such as zero-lag tracking of a periodic target [41] or pursuit maintenance during target occlusion [39]. More recently an effort has been made to provide a more general theoretical framework, which is based on Bayesian inference and the reliability-based cue combination. Although the mapping of this theoretical framework onto neuronal functions remains to be elucidated, it has the clear advantage of generality, as for instance, it would encompass all forms of smooth pursuit behavior, including prediction-based and visually-guided tracking [42–45]. Here, we present a model extending this recent theoretical effort to include the adaptivity to a volatile environment.

Minor:
There are few places in the manuscript where the word “prove” is used and it shouldn’t be used. For example, lines 11, 21… please replace/rephrase.
Remove extra space in line 426
Line 630 has a typo: “exploration versus exploration”

Done, thanks. Please see the tracked changes’ PDF that highlights all the changes we have done on the manuscript.
